# MicroRNA-200a-3p Mediates Neuroprotection in Alzheimer-Related Deficits and Attenuates Amyloid-Beta Overproduction and Tau Hyperphosphorylation *via* Coregulating BACE1 and PRKACB

**DOI:** 10.3389/fphar.2019.00806

**Published:** 2019-07-19

**Authors:** Linlin Wang, Jianghong Liu, Qian Wang, Hailun Jiang, Li Zeng, Zhuorong Li, Rui Liu

**Affiliations:** ^1^Institute of Medicinal Biotechnology, Chinese Academy of Medical Science and Peking Union Medical College, Beijing, China; ^2^Institute of Materia Medica, Chinese Academy of Medical Sciences and Peking Union Medical College, Beijing, China; ^3^Department of Neurology, Xuan Wu Hospital, Capital Medical University, Beijing, China; ^4^Department of Biopharmaceutics, Shenyang Pharmaceutical University, Shenyang, China

**Keywords:** Alzheimer’s disease, miR-200a-3p, BACE1, PRKACB, tau protein, apoptosis

## Abstract

Alzheimer’s disease (AD) is characterized by two landmark pathologies, the overproduction of amyloid-beta peptides (Aβ), predominated by the β-amyloid protein precursor cleaving enzyme 1 (BACE1), and hyperphosphorylation of the microtubule protein, tau, because of an imbalance in a kinase/phosphatase system that involves the activation of the protein kinase A (PKA). Current evidence indicates that brain microRNAs participate in multiple aspects of AD pathology. Here, the role and underlying molecular mechanisms of microRNA-200a-3p (miR-200a-3p) in mediating neuroprotection against AD-related deficits were investigated. The expression of miR-200a-3p was measured in the hippocampus of APP/PS1 and SAMP8 mice and in an AD cell model *in vitro*, as well as in blood plasma extracted from AD patients. The targets of miR-200a-3p were determined using bioinformatics and dual-luciferase assay analyses. In addition, cell apoptosis was detected using flow cytometry, and related protein levels were measured using Western blot and enzyme-linked immunosorbent assay (ELISA) techniques. miR-200a-3p was confirmed to be depressed in microarray miRNA profile analysis *in vitro* and *in vivo*, suggesting that miR-200a-3p is a potential biomarker of AD. Subsequently, miR-200a-3p was demonstrated to inhibit cell apoptosis accompanied by the inactivation of the Bax/caspase-3 axis and downregulation of Aβ_1-42_ and tau phosphorylation levels *in vitro*. Further mechanistic studies revealed that miR-200a-3p reduced the production of Aβ_1-42_ and decreased hyperphosphorylation of tau by regulating the protein translocation of *BACE1* and the protein kinase cAMP-activated catalytic subunit beta (*PRKACB*) associated with the three prime untranslated regions, respectively. Importantly, the function of miR-200a-3p was reversed by overexpression of BACE1 or PRKACB in cultured cells. This resulted in an elevation in cell apoptosis and increases in Aβ_1-42_ and tau hyperphosphorylation levels, involving the epitopes threonine 205 and serine 202, 214, 396, and 356, the favorable phosphorylated sites of PKA. In conclusion, our study suggests that miR-200a-3p is implicated in the pathology of AD, exerting neuroprotective effects against Aβ-induced toxicity by two possible mechanisms: one involving the inhibition of Aβ overproduction *via* suppression of the expression of BACE1 and synergistically decreasing the hyperphosphorylation of tau *via* attenuation of the expression of PKA.

## Introduction

Alzheimer’s disease (AD) is the most common form of dementia affecting people aged 65 and older, which is characterized by two landmark pathologies, extracellular senile plaques consisting of amyloid-beta peptides (Aβ) and intracellular neurofibrillary tangles (NFTs) composed of hyperphosphorylated tau proteins (Ulep et al., 2018). AD has a long preclinical latency and is difficult to diagnose and prevent at early stages. Currently, there are no effective drugs or treatment modalities to stop or reverse the progression of the disease process. Thus, it is of vital importance to explore the underlying mechanisms and potential targets of AD.

Among the complex etiology of AD, Aβ peptides are derived from the successive cleavage of the amyloid protein precursor (APP) by the β-APP cleaving enzyme 1 (BACE1), or β-secretase, and gamma (γ)-secretase enzyme to form Aβ aggregates that have the potential to develop into Aβ plaques (Li et al., 2000). Similarly, the abnormally hyperphosphorylated tau protein in NFTs is thought to be generated from an imbalance in the kinase/phosphatase system, indicated by a series of activity-altered enzymes involving cyclic AMP (cAMP)-dependent protein kinase/protein kinase A (PKA), cyclin-dependent protein kinase 5 (CDK5), glycogen synthase kinase 3β (GSK3β), protein phosphatase 1 (PP1), and protein phosphatase 2A (PP2A), which are key players in the progression of AD (Wang et al., 2014). Furthermore, other pathological factors associated with AD development include oxidative imbalance, neuroinflammation, and calcium homeostasis disturbance, which lead to an overproduction of Aβ peptides by activating BACE1 *via* a set of tau-associated phosphorylated kinases (Chami and Checler, 2012). Considering key AD pathogenic mechanisms, simultaneous interference with two or more causes associated with Aβ-induced tau hyperphosphorylation may achieve better therapeutic efficacy with multiple benefits by combining collaborative mechanisms.

MicroRNAs (miRNAs) are short, single-stranded RNAs of about 20–25 base pairs (bp) in length, which regulate posttranscriptional expression of target messenger RNA (mRNA) by associating with the mRNA three prime-untranslated region (3’-UTR) (Geekiyanage et al., 2012). In addition, miRNA expression has been proven to have tissue, cell, and disease specificity (Ratnadiwakara et al., 2018). Abundance of miRNAs has been illustrated specifically in gene expression changes related to AD and can be also found in the cerebrospinal fluid (CSF) and blood plasma (Fransquet and Ryan, 2018; Zendjabil, 2018). Thus, miRNAs are excellent candidates as noninvasive biomarkers and potential regulators of associated target genes in AD.

MicroRNA-200a-3p (miR-200a-3p), belonging to the miR-200 family of miRNAs and located on chromosome 1p36, plays an important role in human cancers and modulates cell apoptosis and proliferation (Feng et al., 2014; Wu et al., 2017). Accumulating evidence is available illustrating that miR-200a-3p may be involved in AD pathology; however, there is evident controversy about miR-200a-3p levels detected in different Alzheimer’s models. Some studies elucidated that miR-200a-3p was downregulated in K595N/M596L (APPswe)/presenilin 1 (PS1) deltaE9 (APP/PS1) mice during the progression of AD (Liu et al., 2014), whereas some other experiments showed that in the brain of AD patients and in the hippocampus of APP/PS1 mice, the expression of miR-200a-3p was increased (Lau et al., 2013; Zhang et al., 2017). In line with the finding that miR-200a-3p was downregulated in AD, miR-200a-3p was shown to inhibit apoptosis in the SH-SY5Y neuroblastoma cell line *via* the modulation of one of the targets of sirtuin-1 (Salimian et al., 2018). Nevertheless, miR-200a-3p appears to have multiple downstream targets, characterized by the aberrant expression involved in the gene regulation networks among diseases; therefore, the specific roles and underlying molecular mechanisms of miR-200a-3p in AD remains unexplored.

In this study, we investigated the expression of miR-200a-3p in the hippocampus of APP/PS1 and senescence-accelerated mouse prone 8 (SAMP8) mice and in an AD cell model *in vitro*, as well as in blood plasma extracted from AD patients. We further explored the roles of miR-200a-3p and its potential molecular mechanisms in the AD cell model. Collectively, this study revealed that miR-200a-3p supplementation might play a neuroprotective role in AD, which highlights potential future research avenues and novel therapeutic targets for AD.

## Materials and Methods

### Animals and Treatments

APP/PS1 mice and age-matched wild-type (WT) littermates were purchased from the Jackson Laboratory (Bar Harbor, ME). SAMP8 and senescence-accelerated mouse resistance 1 (SAMR1) mice were provided by the Institute of Genetics and Developmental Biology of the Chinese Academy of Sciences. The animals had *ad libitum* access to food and water at stable room temperature and humidity environment according to the Guide for the Care and Use of Laboratory Animals. The experiment was approved by the ethical committee of the Institute of Medicinal Biotechnology (IMB-D8-2018071102).

The mice were then divided into the following groups: 1-month-old, 3-month-old, 6-month-old, or 9-month-old mice, with the inclusion of age-matched control mice (WT or SAMR1). Each group was composed of four mice (two males and two females per group). Depending on the different ages of each group, brains were collected and then evaluated by real-time polymerase chain reaction (PCR) analysis during the course of the disease.

### Human Blood Sample Data and Collection

Blood samples of seven AD patients and five normal age-matched volunteers (NAVs) were acquired from the Xuanwu Hospital Capital Medical University, and the study was approved by the ethics committee of Xuanwu Hospital Capital Medical University, China (**Table 1**). The peripheral blood was collected from each patient after fasting for 12 h. The serum was separated by centrifugation at 1,000 × g for 10 min at room temperature, followed by centrifugation at 130,000 × g for 5 min at 4°C. The samples were stored at 80°C until required.

**Table 1:** Clinical data of AD patients compared to normal age-matched volunteers (NAVs).

Group	Cases (n)	Age	Gender
NAVs	5	67.60 ± 2.65	2 M/3 F
AD	7	77.14 ± 8.33	3 M/4 F

NAVs, normal age-matched volunteers; AD, AD patients; M, males; F, females.

### Cell Culture and Plasmid Transfection

Human neuroblastoma SH-SY5Y cells (ATCC; Manassas, VA) and human embryonic kidney (HEK)293 cells (ATCC) were maintained in Dulbecco’s modified Eagle’s medium (DMEM) supplemented with 10% fetal bovine serum (FBS) (Gibco/Invitrogen, Grand Island, NY) at 37°C in a humidified 5% CO_2_ incubator. SH-SY5Y cells transfected with the Swedish mutant form of human APP (referred to as “APPswe cells”) is an established AD cell model, in which copper can trigger the neurotoxicity of Aβ, leading to cell apoptosis (Liu et al., 2012; Zhao et al., 2013). APPswe cells were then cultured in DMEM/F-12 supplemented with 10% FBS and 500 μg/ml G418 (Invitrogen). miR-200a-3p mimics, miR-200a-3p inhibitor, and respective negative controls (NCM or NCI), as well as BACE1-siRNA, PRKACB-siRNA, and respective NCs were synthesized by GenePharma (Shanghai, China). The oligonucleotides were transfected in a final concentration of 50 nM using the Lipofectamine 3000 reagent (Invitrogen; Carlsbad, CA). The BACE1 and PRKACB expression vectors were constructed by inserting human BACE1 and PRKACB cDNA into pCMV6 vector, and their promoters were CMV and tagged with Myc-DDK. The pCMV6-BACE1-Myc-DDK and pCMV6-PRKACB-Myc-DDK were purchased from ORIGENE (Beijing, China) and transfected into APPswe cells in a final concentration of 2 μg/ml using the Lipofectamine 3000 reagent.

### Quantitative Reverse Transcription Polymerase Chain Reaction Analysis

The total RNA of neuronal cells and mouse brain tissue were extracted using TRIZOL (Invitrogen) according to the manufacturer’s instructions. The TaqMan microRNA Reverse Transcription reagent (Invitrogen) was used to reverse transcript 10 ng of total RNA to complementary DNA (cDNA). The following reactions were performed in a total volume of 20 μl containing the following: 1 μl TaqMan small RNA assay, 1.3 μl cDNA sample, 10 μl TaqMan universal PCR Master Mix, and 10 μl nuclease-free water. Each sample was run in triplicate. Small nuclear RNA U6 was used as normalization. The thermo cycle conditions were set as follows: enzyme activation at 50°C for 2 min, denaturation at 95°C for 10 min, followed by 40 cycles of denaturation at 95°C for 15 s and extension at 60°C for 1 min. For mRNA analysis, 2 μg of total RNA was mixed with Maxima H Minus cDNA Synthesis Master Mix (Invitrogen) for reverse transcription. Subsequently, a qPCR assay was used using the SYBR Green Master Mix (Invitrogen) on an ABI-7500 Fast Real-Time PCR System (Applied Biosystems, Foster City, CA). The primers used are listed in **Table 2**. The data were analyzed using the 2-ΔΔCT method.

**Table 2:** PCR primer sequences.

Primer Name	Primer Sequence
BACE1-F	5’-CCGGCGGGAGTGGTATTATG-3’
BACE1-R	5’-GCAAACGAAGGTTGGTGGT-3’
PRKACB-F	5’-CCATGCACGGTTCTATGCAG-3’
PRKACB-R	5’-GTCTGTGACCTGGATATAGCCTT-3’
β-actin-F	5’-CATGTACGTTGCTATCCAGGC-3’
β-actin-R	5’-CTCCTTAATGTCACGCACGAT-3’

F, forward; R, reverse.

### Dual-Luciferase Reporter Assay

The 3’-UTR of *BACE1* and *PRKACB* containing the binding site of miR-200a-3p were cloned into the luciferase reporter plasmid (Promega; Madison, WI), and the binding site mutants were synthesized by a commercial company (Sangon Biotechnology, Shanghai, China). The WT or mutant luciferase plasmid together with the pRT-TK Renilla luciferase vector (Promega) were cotransfected with miR-200a-3p mimics or NCs into HEK293 cells. After 48 h, the corresponding vector’s luminescence was detected using the GloMax Multi luminometer (Promega) with a Dual-Luciferase Reporter Assay system. The Renilla luminescence was used to normalize the signal. All of the experiments were repeated four times independently.

### Western Blotting Analysis

Protein samples were extracted from differently treated cells using the M-PER Mammalian protein Extraction Reagent (Pierce Biotechnology; Rockford, IL) at appropriate time points, according to the manufacturer’s instructions. Then, sodium dodecyl sulfate polyacrylamide gel electrophoresis (SDS-PAGE) was used to separate target proteins and were subsequently transferred to a polyvinylidene fluoride (PVDF) membrane (Merck Millipore, Billerica, MA). The membranes were then blocked in 5% nonfat milk dissolved in tris-buffered saline (TBS) containing 0.1% Tween-20 (TBST) for 1 h and then probed with the corresponding antibody as listed in **Table 3** overnight at 4°C. The next day, membranes were washed three times with TBST and incubated with the appropriate secondary antibodies (Abcam, Cambridge, MA) for 1 h at room temperature. The protein bands were visualized and quantified by a protein imaging system (Biorad, Munich, Germany).

**Table 3:** Primary antibodies used in this study.

Antibody	Type	Diluted	Source
Anti-BACE1	Monoclonal	1:1,000	Abcam
Anti-PRKACB	Monoclonal	1:1,000	Abcam
Anti-AT8	Monoclonal	1:1,000	Abcam
Anti-pTS396-Tau	Monoclonal	1:500	Abcam
Anti-pTS214-Tau	Monoclonal	1:500	Abcam
Anti-pTS356-Tau	Monoclonal	1:500	Abcam
Anti-tau	Monoclonal	1:500	Abcam
Anti-caspase-3	Monoclonal	1:1,000	Abcam
Anti-Bax	Monoclonal	1:1,000	Abcam
Anti-GAPDH	Monoclonal	1:1,000	Abcam

### Aβ_1-42_ Assay

APPswe cells were seeded in 24-well plates and transfected with miR-200a-3p, BACE1, or PRKACB related plasmids. After 48 h, cells were lysed and detected using the Human Aβ_1-42_ enzyme-linked immunosorbent assay (ELISA)Kit (Invitrogen) according to the manufacturer’s instructions. The concentration of Aβ_1-42_ was determined by the absorbance value detected using a microplate reader (Tecan Group Ltd., Mannedorf, Switzerland) at 450 nm.

### Cell Apoptosis Assay by Flow Cytometry

APPswe cells were transfected with miR-200a-3p mimics, miR-200a-3p inhibitor, pCMV6-BACE1-Myc-DDK, pCMV6-PRKACB-Myc-DDK, BACE1 siRNA, PRKACB siRNA, and their relative controls separately or in combination. After transfection, copper was added to trigger the Aβ toxicity. The Annexin BD Pharmingen FITC-Annexin V/propidium iodide (PI) Apoptosis Detection Kit (BD Biosciences, San Jose, CA) was used to detect cell apoptosis of APPswe cells after different plasmid transfections, according to the manufacturer’s protocol. Briefly, cells were washed in cold phosphate buffer saline (PBS) and stained with a mixture of FITC-labeled Annexin-V and PI on ice for 15 min and analyzed in a FACSCalibur flow cytometry (BD Biosciences).

### Caspase-3 Activity Assay

APPswe cells were transfected with miR-200a-3p mimics, miR-200a-3p inhibitor, and the relative controls, as well as pCMV6-BACE1-Myc-DDK and pCMV6-PRKACB-Myc-DDK separately or in combination. After different treatments, APPswe cells were used to detect the activity of caspase-3 using the Human Active Caspase-3 (Asp175) SimpleStep ELISA Kit (Abcam, Cambridge, MA) according to the manufacturer’s instructions.

### Bioinformatics and Heatmap Analysis

The miR-200a-3p target predicted by computer-aided algorithms was obtained from TargetScan. The miRNA expression profiling was performed as described before (Wang et al., 2017). RNA extraction was performed with TRIzol regent and synthesized into cDNA. After purification, the cDNA was labeled with Hy3/Hy5 and hybridized to the microarray (Exiqon, Vedbæk, Denmark) according to the Exiqon’s instruction. The slides were scanned by Axon GenePix 4000B microarray scanner (Exiqon), and heatmap analysis of the expression of the selected miRNAs at different stages of AD development was accomplished using the color gradation function of Microsoft Excel 2016 (Microsoft Corporation, Redmond, WA).

### Statistical Analysis

Data are represented as mean ± standard error of the mean (SEM). All of the experiments were repeated at last three times. Data were analyzed using Student’s t-test or one-way ANOVA followed by Tukey’s *post hoc* tests where appropriate. Comparisons between two groups were performed using Student’s t-test. *P* values of less than 0.05 were considered statistically significant. All of the analyses were performed using the GraphPad Prism Version 7.0 (GraphPad Prism Software, La Jolla, CA).

## Results

### The Expression of miR-200a-3p is Decreased During AD Progression

**Figure 1:**
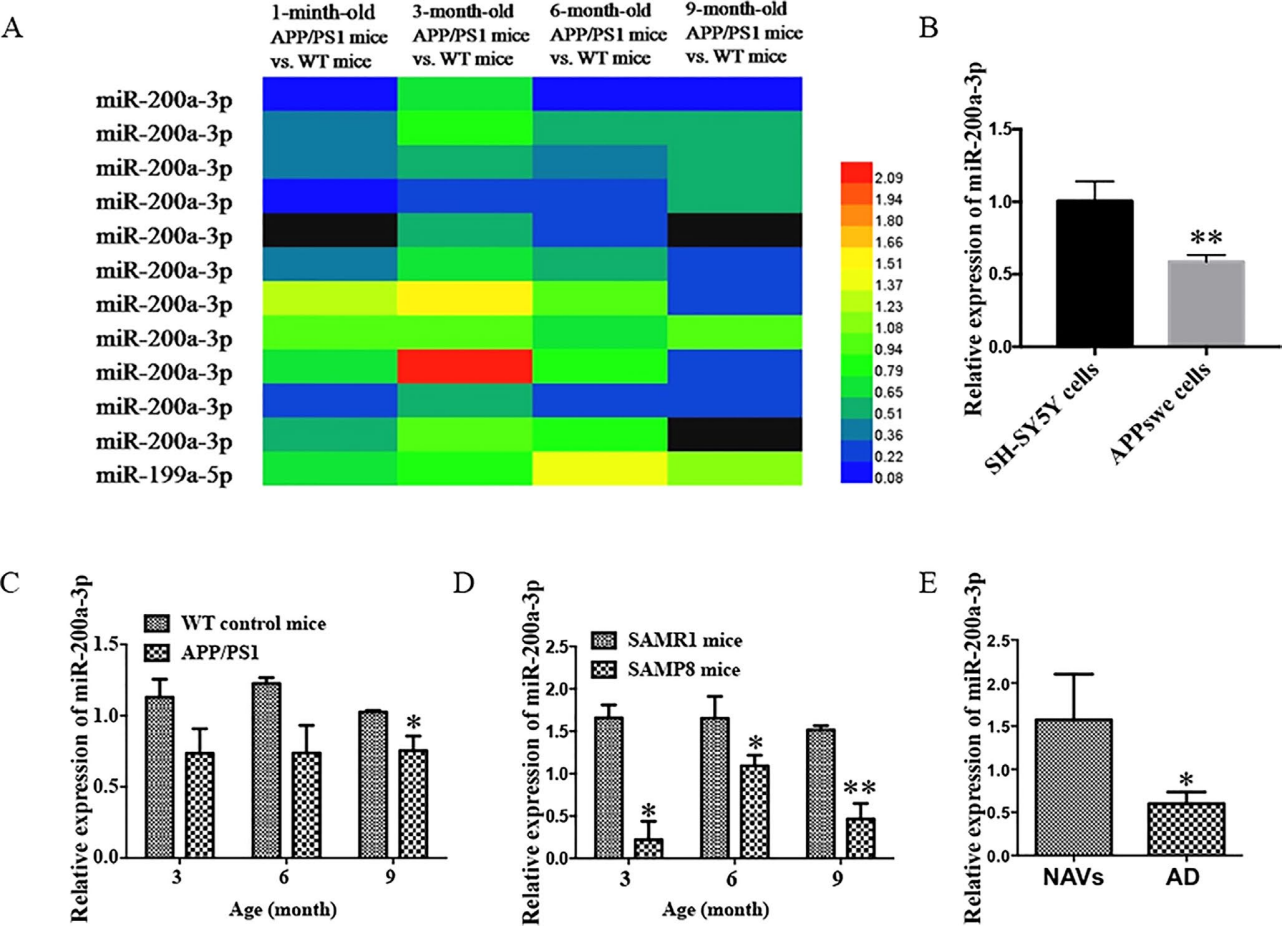
Aberrant expression of miR-200a-3p is involved in the progression of AD. **(A)** Expression of miR-200a-3p in APP/PS1 mouse brains using microarray analysis at different stages of the disease process. **(B)** Decreased expression of miR-200a-3p in APPswe cells compared with normally cultured SH-SY5Y cells (*n* = 3). **(C**, **D)** Decreased expression of miR-200a-3p in the hippocampus of APP/PS1 **(C)** and SAMP8 mice **(D)** (*n* = 4). **(E)** Reduced levels of miR-200a-3p in the plasma of AD patients compared with normal age-matched volunteers (NAVs) (*n* = 5–7). Data are shown as the mean ± SEM.*P < 0.05. **P < 0.01 versus relevant control.

It has been described that the APP/PS1 double transgenic mouse model develops AD pathology and cognitive impairment with increasing age (Trinchese et al., 2004). Before exploring the role of miR-200a-3p in the pathological processes of AD, we determined the manifestation of miR-200a-3p in miRNA profiles of APP/PS1 transgenic mice using microarray analysis. As shown in **Figure 1A**, the levels of miR-200a-3p were significantly downregulated in 6-month-old and 9-month-old mice. Moreover, to assess the changes of miR-200a-3p expression during AD progression, SH-SY5Y cells overexpressing the APPswe plasmid and two mouse models, the APP/PS1 transgenic and the mutant SAMP8 strains, were used because they have been previously shown to express deficits closely similar to AD (Ding et al., 2008; Liu et al., 2012; Takagane et al., 2015). Our results demonstrated that the expression of miR-200a-3p was significantly reduced in APPswe cells when compared to normally cultured SH-SY5Y cells (**Figure 1B**, *P* < 0.01). This was in line with the depressed levels of miR-200a-3p found in the hippocampus of APP/PS1 and SAMP8 mice at 3, 6, or 9 months of age when compared to their corresponding WT control counterparts (**Figures 1C, D**, *P* < 0.05–0.01). Because miRNAs can circulate in the blood and the CSF, they are attractive candidates as biomarkers of AD. To this end, we explored the expression of miR-200a-3p in the plasma of AD patients and NAVs and found that miR-200a-3p levels were significantly downregulated in the blood plasma of AD patients when compared to those in NAVs (**Figure 1E**, *P* < 0.05). Therefore, these data confirmed a reduced tendency of miR-200a-3p expression levels in the pathological processes of AD and also indicated that miR-200a-3p might participate in the regulation of this process.

### miR-200a-3p Inhibits Aβ_1-42_ Production, Attenuates Tau Phosphorylation, and Suppresses Apoptosis in APPswe Cells

**Figure 2:**
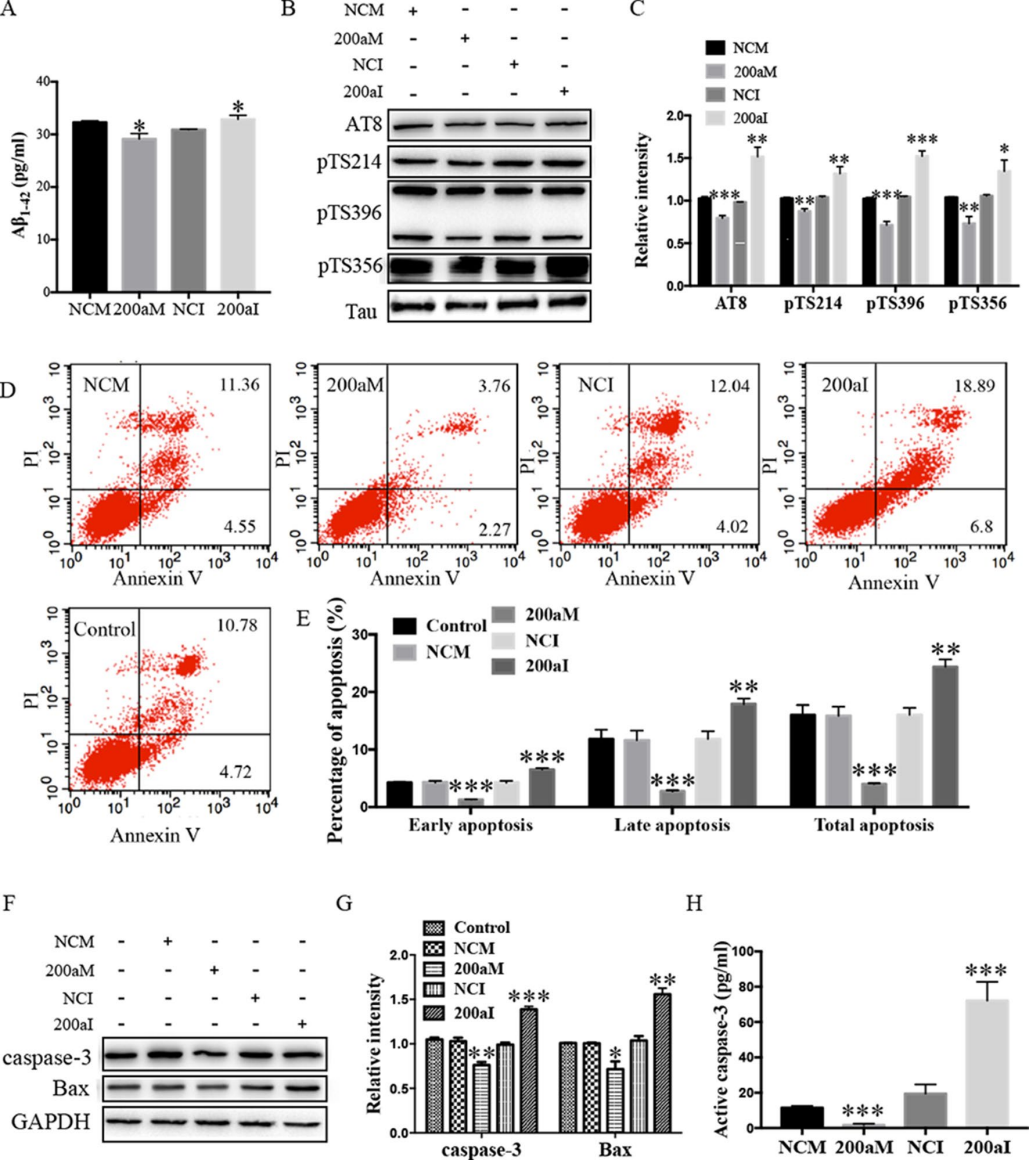
The activity of miR-200a-3p exhibits neuroprotective properties in APPswe cells. **(A)** miR-200a-3p mimics (200aM) inhibited the production of Aβ_1-42_ in APPswe cells whereas miR-200a-3p inhibitor (200aI) increased Aβ_1-42_ production (*n* = 4). **(B**, **C)** miR-200a-3p mimics (200aM) decreased the phosphorylated levels of tau at Ser202/Thr205 (AT8), Ser214 (pTS214), Ser396 (pTS396), and Ser356 (pTS356) sites, as demonstrated here with representative images **(B)** and quantitative analysis **(C)** by Western blotting (*n* = 3). **(D**, **E)** Quantification of cell apoptosis in the presence of miRNA-200a-3p mimics (200aM), miRNA-200a-3p inhibitors (200aI), corresponding negative controls (NCM/NCI), and nontransfected controls (control) in APPswe cells, as demonstrated by representative images **(D)** and quantitative analysis **(E)** by flow cytometry (*n* = 3). **(F**, **G)** miR-200a-3p mimics decreased the expression of caspase-3 and Bax, as demonstrated here by representative images **(F)** and quantitative analysis **(G)** by Western blotting. **(H)** miR-200a-3p mimics decreased the activity of caspase-3 in APPswe cells (*n* = 4). Data are shown as the mean ± SEM. *P < 0.05, **P < 0.01, ***P < 0.001 versus relative control.

Aβ_1-42_ peptides, derived *via* processing of APP by BACE1, contribute to AD pathogenesis (Li et al., 2000). We determined the effect of miR-200a-3p on Aβ_1-42_ production and found that overexpression of miR-200a-3p inhibited the production of Aβ_1-42_ in miR-200a-3p mimics-transfected APPswe cells, whereas miR-200a-3p knockdown led to an Aβ_1-42_ overproduction in miR-200a-3p inhibitor-transfected APPswe cells (**Figure 2A**, *P* < 0.05).

The hyperphosphorylation of tau is associated with neural apoptosis and plays an important role in the development of AD. When we overexpressed miR-200a-3p in APPswe cells, the phosphorylation of tau protein at serine 202/threonine 205 (AT8), serine 214 (S214), serine 396 (S396), and serine 356 (S356) epitopes was significantly decreased (**Figures 2B, C**, *P* < 0.01–0.001), whereas the inhibition of miR-200a-3p led to opposite effects (*P* < 0.05–0.001).

Furthermore, flow cytometry using PI and Annexin V staining was performed to analyze the involvement of apoptotic pathways in APPswe cells transfected with miR-200a-3p mimics, miR-200a-3p inhibitor, or NCs. Our results revealed that the ratios of early apoptosis, late apoptosis, and total apoptosis in APPswe cells overexpressing miR-200a-3p were all significantly decreased (**Figures 2D, E**, all *P* < 0.001), whereas inhibition of miR-200a-3p significantly increased the apoptotic ratios (*P* < 0.01–0.001). Among apoptotic pathways, the expression levels of the proapoptotic protein Bax were found to be downregulated when miR-200a-3p was overexpressed in APPswe cells and changed in an opposite manner when miR-200a-3p was inhibited (**Figures 2F, G**, *P* < 0.01, *P* < 0.001), accompanied by the activity and protein level of caspase-3, which were affected in the same manner in all transfected APPswe cells (**Figures 2F–H**, *P* < 0.05–0.001). The apoptotic ratios, expression of Bax, and activity and protein level of caspase-3 in NC-transfected APPswe cells were not altered compared with those in the non-transfected groups. Collectively, these observations indicated that the upregulation of miR-200a-3p exerted a neuroprotective effect against AD deficits in APPswe cells.

### The *BACE1* mRNA is a Direct Target of miR-200a-3p

**Figure 3:**
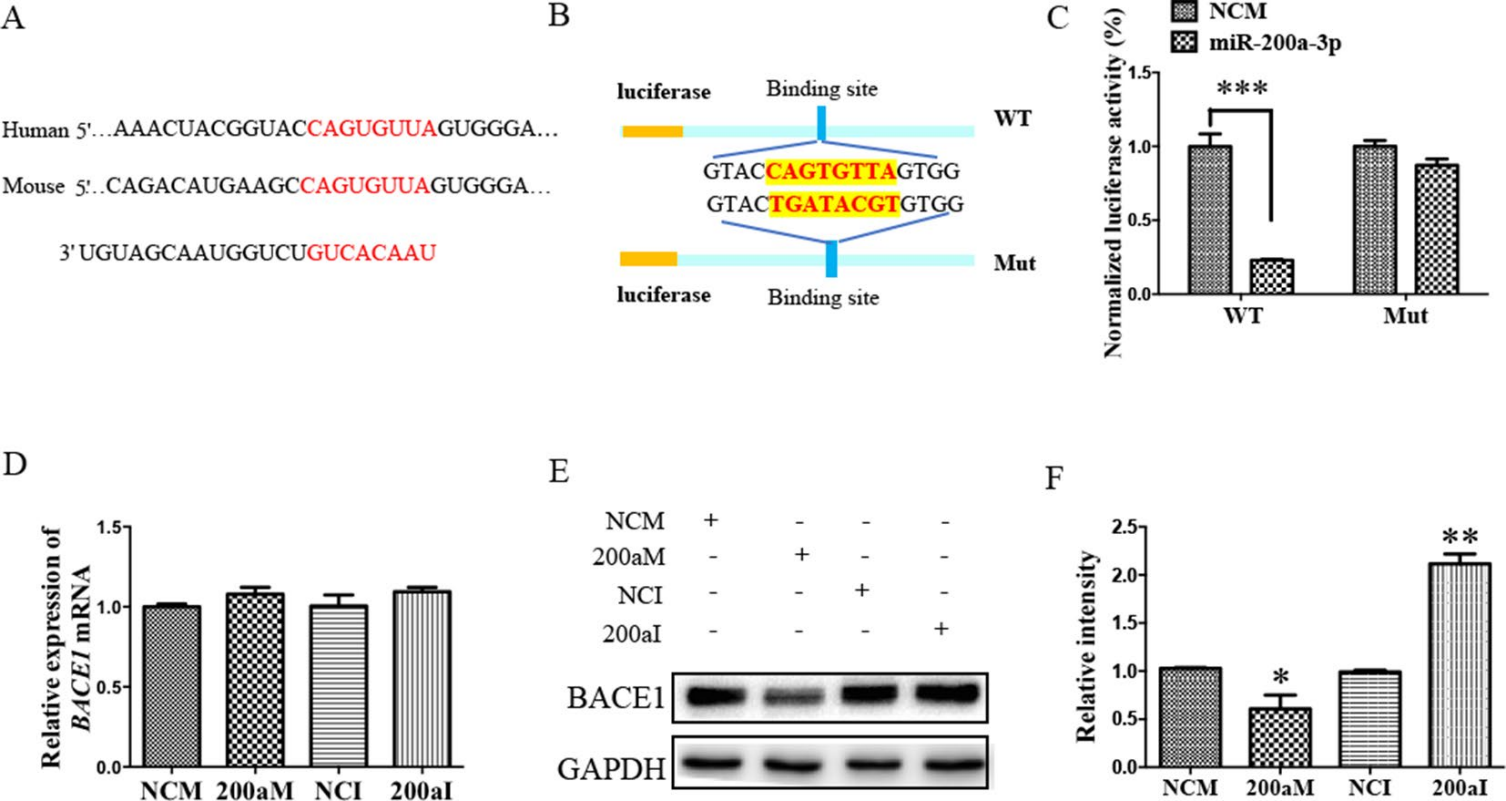
The *BACE1* mRNA is a direct target of miR-200a-3p. **(A)** Bioinformatic analysis predicting the binding site of miR-200a-3p and *BACE1* mRNA. **(B)** Design of the recombinant Luc-*BACE1*-MUT (mutant) and Luc-*BACE1*-WT (wild-type) construction. **(C)** Changes in the relative luciferase activity in each group after plasmids transfection. miRNA-200a-3p mimics caused significant inhibition of reporter luciferase activity in the construct with a wild-type (WT) *BACE1* 3’-UTR in contrast to the mutant (Mut) *BACE1* 3’-UTR in HEK293 cells (*n* = 4). **(D)** Quantification analysis of the levels of *BACE1* mRNA after transfection with miRNA-200a-3p mimics (200aM), miRNA-200a-3p inhibitors (200aI), and corresponding negative controls (NCM/NCI) (*n* = 3). **(E**, **F)** The expression of the BACE1 protein is signiﬁcantly decreased and increased after miR-200a-3p mimics (200aM) and inhibitor (200aI) transfection, respectively, relative to the corresponding negative controls (NCM/NCI), as demonstrated here by representative images **(E)** and quantitative analysis **(F)** by Western blotting (*n* = 3). The data are presented as the mean ± SEM. *P < 0.05, **P < 0.01, ***P < 0.001 versus relevant control.

To investigate the role of miR-200a-3p in AD development, it was necessary to find targets correlated with signaling transduction associated with AD pathology. For this, we first performed computational analyses to identify potential binding sites of miR-200a-3p using the miRNA target prediction database TargetScan. Our results indicated that miR-200a-3p had a potential target site in the 3’-UTR of the *BACE1* mRNA (**Figure 3A**). Subsequently, the systematic diagrams of miR-200a-3p and *BACE1* mRNA 3’-UTR were established (**Figure 3B**).

Furthermore, a dual-luciferase reporter assay was used to investigate the manner in which miR-200a-3p regulated *BACE1*. For this, the 3’-UTR of *BACE1* mRNA containing WT or mutation binding site of miR-200a-3p was cloned in a luciferase vector, and miR-200a-3p mimics or NCs together with Renilla plasmid were cotransfected into HEK293 cells. The luminescence activity was significantly decreased in the cells that were cotransfected with miR-200a-3p mimics plus *BACE1* mRNA 3′-UTR WT (**Figure 3C**, *P* < 0.001). However, there was no effect on the luciferase activity in the cells cotransfected with luciferase plasmids containing mutation binding site of miR-200a-3p. Thus, we concluded that miR-200a-3p was specifically binding to the 3’-UTR of *BACE1*.

Because a direct relationship between miR-200a-3p and *BACE1* expression was established, Western blot and qRT-PCR analysis after the transfection of miR-200a-3p mimics, miR-200a-3p inhibitor, or NCs were performed to identify the target function of miR-200a-3p on the *BACE1* gene. As shown in **Figures 3E, F**, overexpression of miR-200a-3p downregulated the expression of the BACE1 protein (*P* < 0.05), whereas the inhibitory expression of miR-200a-3p upregulated BACE1 protein levels (*P* < 0.01). Because of the result that miR-200a-3p mimics or miR-200a-3p inhibitor did not influence the expression of *BACE1*mRNA at the transcriptional level significantly (**Figure 3D**), we concluded that the interaction manner of miR-200a-3p with BACE1 relied on the regulation of the protein translation process, rather than influencing the stability of *BACE1* mRNA.

### The PRKACB is Another Target of miR-200a-3p

**Figure 4:**
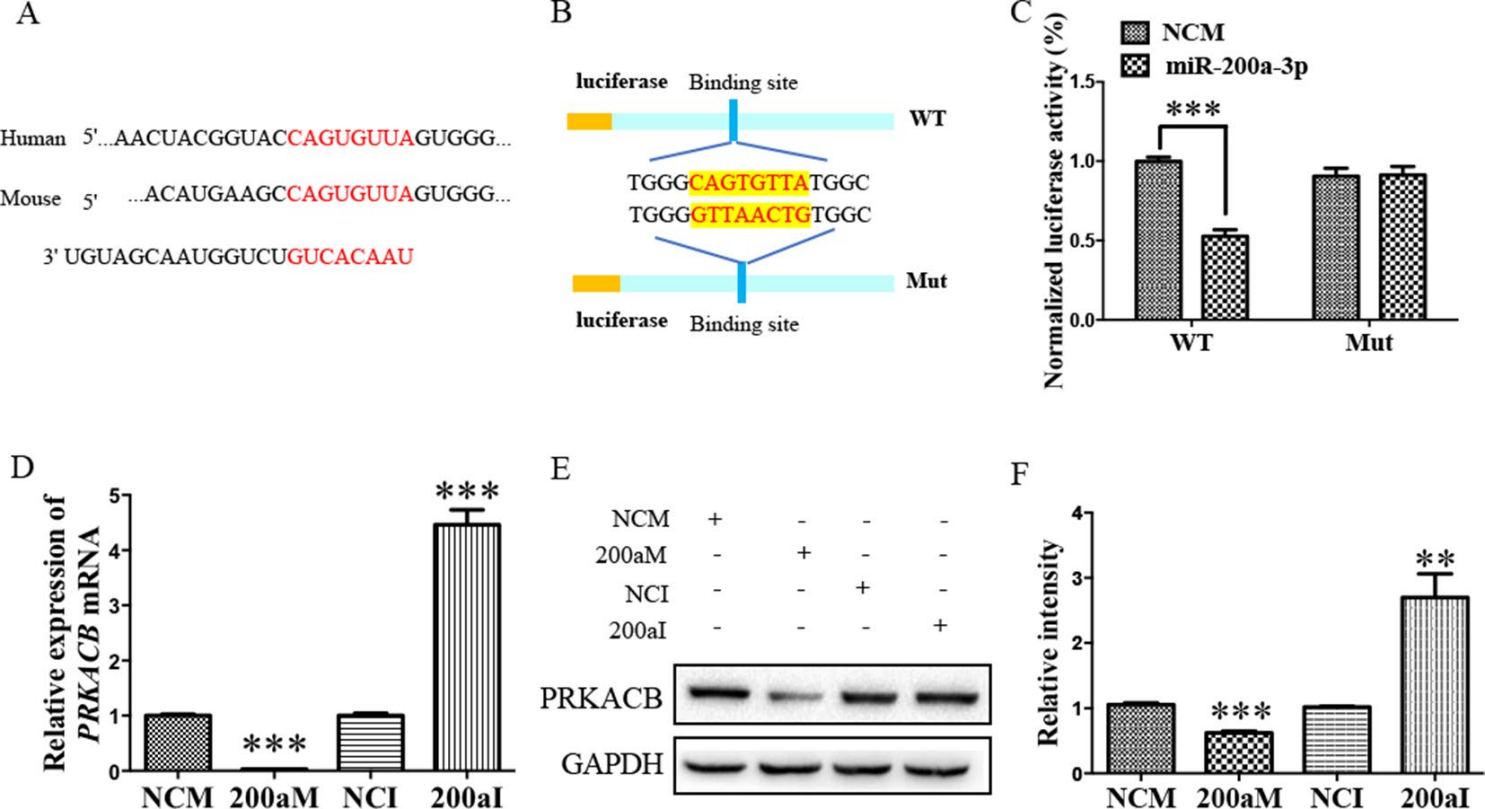
The PRKACB is another target of miR-200a-3p. **(A)** Bioinformatic analysis indicating the binding site of miR-200a-3p with the 3’-UTR of *PRKACB* mRNA. **(B)** Design of the recombinant Luc-*PRKACB*-MUT (mutant) and Luc-*PRKACB*-WT (wild-type) construction. **(C)** Changes in the relative luciferase activity in each group after plasmids transfection. miRNA-200a-3p mimics caused signiﬁcant inhibition of reporter luciferase activity in the construct with a wild-type (WT) *PRKACB* 3’-UTR in contrast to the mutant (Mut) *PRKACB* 3’-UTR in HEK293 cells (*n* = 4). **(D)** Quantification of *PRKACB* mRNA levels after transfection with miRNA-200a-3p mimics (200aM), miRNA-200a-3p inhibitors (200aI), and corresponding negative controls (NCM/NCI) (n = 3). **(E**, **F)** The expression of the PRKACB protein signiﬁcantly decreases and increases after miRNA-200a-3p mimics (200aM) and inhibitor (200aI) transfection, respectively, relative to the corresponding negative controls (NCM/NCI), as demonstrated here by representative images **(E)** and quantitative analysis **(F)** by Western blotting (*n* = 3). Data are shown as the mean ± SEM. *P < 0.05, **P < 0.01, ***P < 0.001 versus relevant control.

Our bioinformatic analysis predicted that there was another candidate target of miR-200a-3p, *PRKACB*, a universally conserved gene that encodes one of the paralogous catalytic subunits of PKA that increases the levels of phosphorylated tau at Ser214, Ser356, and Ser396 epitopes by a number of kinases, widely reported in AD brains and mouse models (Wang et al., 2013; Wang et al., 2015). Importantly, the 3’-UTR of the *PRKACB* transcript contains a putative miR-200a-3p binding site (**Figure 4A**). Subsequently, the luciferase reporters were constructed containing the WT or binding site mutations of *PRKACB* 3’-UTR (**Figure 4B**). Our results indicated that transfection of miR-200a-3p mimics significantly inhibited the activity of the luciferase reporter for WT 3’-UTR *PRKACB* (**Figure 4C**, *P* < 0.001), but not for binding site mutations of *PRKACB* 3’-UTR, indicating the potential interaction between miR-200a-3p and *PRKACB*. We further examined the regulatory manner of miR-200a-3p in PRKACB at the protein and mRNA levels in APPswe cells by transfecting miR-200a-3p mimics and miR-200a-3p inhibitors using Western blot and qPCR analysis. Our data illustrated that miR-200a-3p mimics significantly downregulated both the mRNA and protein expressions of PRKACB (**Figures 4D–F**, both *P* < 0.001), whereas miR-200a-3p inhibitors upregulated PRKACB at these two levels (*P* < 0.001 and *P* < 0.01). Therefore, we suggested that PRKACB may act as another target of miR-200a-3p by associating with the 3’-UTR of *PRKACB*.

### miR-200a-3p Displays a Neuroprotective Role in APPswe Cells by Regulating BACE1 and PRKACB, Decreasing Aβ Overproduction and tau Hyperphosphorylation, Respectively

**Figure 5:**
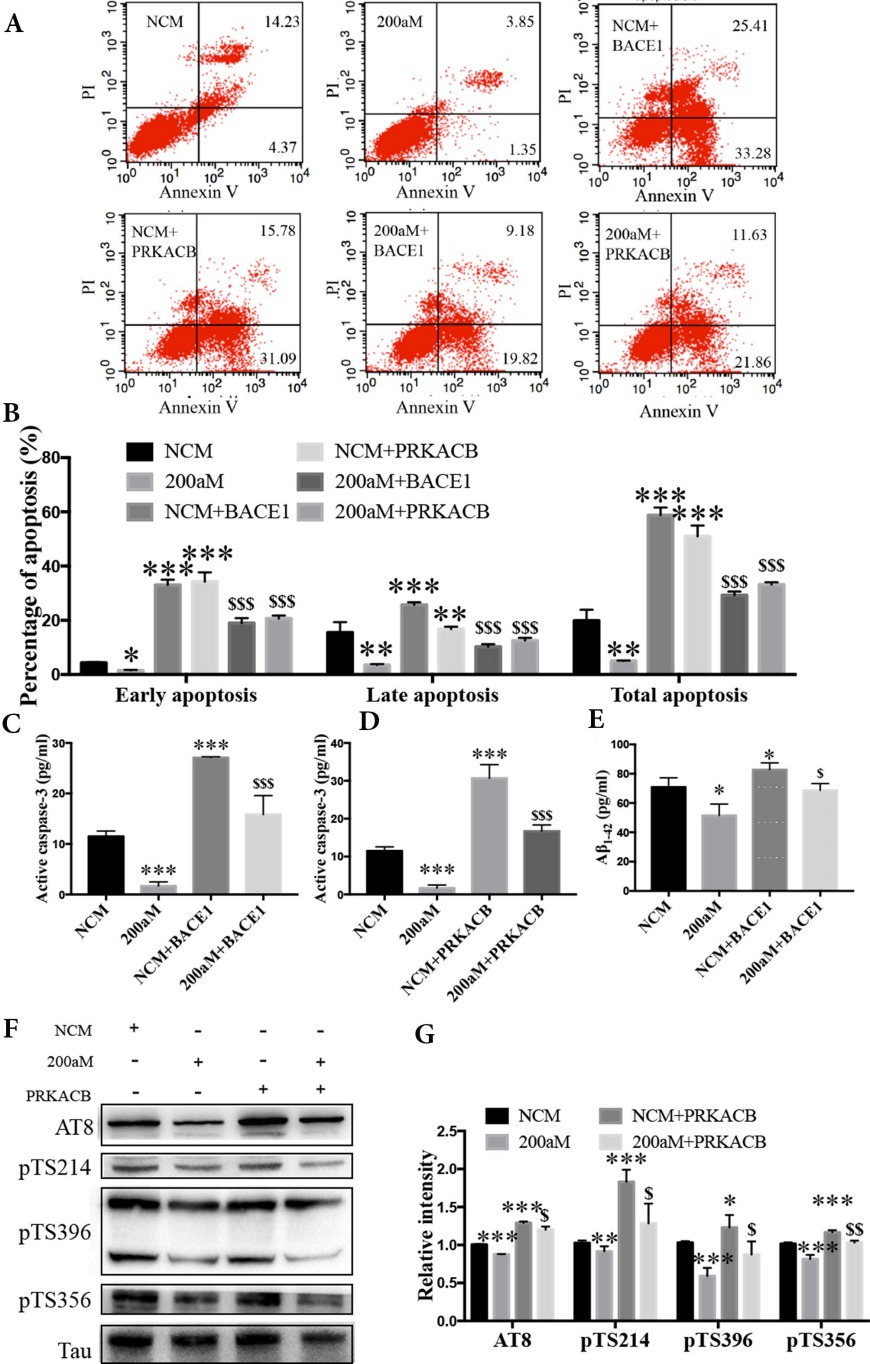
Neuroprotection of miR-200a-3p by regulating the participation of BACE1 and PRKACB in Aβ overproduction and tau hyperphosphorylation, respectively, in APPswe cells. **(A**, **B)** Effects of miR-200a-3p, BACE1, and PRKACB overexpression on apoptosis of APPswe cells detected using flow cytometry assay **(A)** and demonstrated by quantitative analysis **(B)** (*n* = 3). **(C**, **D)** The release of active caspase-3 in APPswe cells when miR-200a-3p and BACE1 **(C)** or PRKACB **(D)** overexpression measured using ELISA (*n* = 4). **(E)** Effects of miR-200a-3p and BACE1 overexpression on the production of Aβ_1-42_ peptides in a cell model of AD (*n* = 4). **(F, G)** Effects of miR-200a-3p and PRKACB overexpression on tau phosphorylation at AT8, Ser214 (pTS214), Ser396 (pTS396), and Ser356 (pTS356) sites in APPswe cells, as demonstrated by representative images **(F)** and quantitative analysis **(G)** by Western blotting (*n* = 3). Data are shown as the mean ± SEM. *P < 0.05, **P < 0.01, ***P < 0.001 versus NCM. *^$^* *P* < 0.05, *^$$^* *P* < 0.01, *^$$$^* *P* < 0.001 versus 200aM.

During our study of miR-200a-3p on the pathological changes in AD associated with BACE1 and PRKACB, we explored the cell apoptosis, levels of Aβ_1-42_, and phosphorylation of the tau protein at several phosphorylated sites that PKA favors after overexpression of BACE1 and PRKACB cotransfected with miR-200a-3p mimics into APPswe cells. Apoptosis-related assays indicated that cotransfection of BACE1 and PRKACB with NCM increased the early, late, and total apoptotic ratios of APPswe cells and the activity of caspase-3, an apoptotic marker, as well (**Figures 5A–D**, *P* < 0.01–0.001). Meanwhile, the neuroprotection of miR-200a-3p mimics involving the suppression of apoptotic ratios and inhibition of active caspase-3 release in APPswe cells (*P* < 0.05–0.001) was reversed *via* cotransfection with BACE1 and PRKACB, respectively (*P* < 0.05–0.001). Additionally, when BACE1 and PRKACB were knockdown using BACE1 siRNA and PRKACB siRNA, a decreased tendency of miR-200a-3p neuroprotection was seen in APPswe cells (**Supplementary Figure 1**). These results indicated that there might be a direct neuroprotective relationship between miR-200a-3p and BACE1 and PRKACB.

As for Aβ overproduction and tau hyperphosphorylation, cotransfection of BACE1 and PRKACB with NCM increased the production of Aβ_1-42_ and the expression of tau phosphorylation at AT8, Ser214, Ser396, and Ser356 (**Figures 5E–G**, *P* < 0.05–0.001). Importantly, cotransfection of BACE1 and PRKACB together with miR-200a-3p mimics abolished the beneficial effects of miR-200a-3p, increasing Aβ_1-42_ production and enhancing the levels of tau phosphorylation at detected phosphorylated sites (*P* < 0.05–0.01). Collectively, these findings suggest that miR-200a-3p may protect neural cells by coregulating BACE1 and PRKACB *in vitro*.

## Discussion

AD is a complex neurodegenerative disease affected by multigene activity and layers, which to date has no effective therapeutic treatments. As a result, a multigene regulatory approach is sought out as an AD therapeutic modality. Markedly, miRNAs are considered to be attractive candidates for their modulatory role in mRNA transcription and protein translation. In the present study, we deduced three primary findings regarding the function of one identified miRNA and its role in the pathological progression of AD. First, miR-200a-3p was found to demonstrate the consistent depression observed in the brain tissue of AD mice, in cell culture models of AD, and in the blood of AD patients. Second, miR-200a-3p was verified as a participant in the pathogenesis of AD directly *via* the regulation of *BACE1* and *PRKACB* expression levels using a target prediction database and through a dual-luciferase reporter assay. Third, miR-200a-3p was found to have neuroprotective effects by suppressing the overproduction of Aβ and the hyperphosphorylation of the tau protein *via* regulating the protein expression of BACE1 and PRKACB in the AD cell model, respectively. Here, we provide an alternative strategy by targeting miR-200a-3p for the prevention and/or treatment of AD.

Although AD develops as a multifactorial process, the abnormal overproduction and accumulation of Aβ are still considered to be key events (Moore et al., 2014). BACE1 is highly expressed in neurons and is a crucial protease that functions in the first step of the pathway leading to the majority of the Aβ production in the pathology of AD (Marques et al., 2012). BACE1 has been proposed as a viable therapeutic target for AD; however, challenges with the currently investigated BACE1 inhibitors, involving low oral bioavailability, long serum half-life, and low blood–brain barrier (BBB) penetration ratio (Vassar, 2014), have not been fully overcome yet. Thus, investigators have turned toward miRNA replacement therapy in light of their specific interactions. There are several miRNAs that have been proven to target BACE1, such as miR-124, miR-29c, and miR-195 (Zong et al., 2011; Fang et al., 2012; Zhu et al., 2012; Du et al., 2017). Following deep data mining analysis combined with target prediction and confirmatory experiments, we found that the downregulation of miR-200a-3p was implicated in AD etiology and targeted the 3’-UTR of BACE1 mRNA. We also verified that there was a negative correlation between miR-200a-3p and BACE1 in the blood of AD patients and that the imposed upregulation of miR-200a-3p significantly downregulated the expression of BACE1 at the protein level. As BACE1 is known to be significantly increased in the brains of AD patients, as well as in their CSF (Stozická et al., 2007; Evin and Hince, 2013), we suggest that miR-200a-3p could act as a potential peripheral biomarker together with BACE1 for AD diagnosis and/or treatment.

miR-200a-3p is a member of the miR-200 family of miRNAs that plays a vital role in various cancers, including bladder cancer, breast cancer, colorectal cancer, and gastric cancer, among others (Feng et al., 2014; Muralidhar and Barbolina, 2015). Recently, the miR-200 family of proteins was found to be crucial for neural differentiation and proliferation, along with their participation in Aβ production in AD (Liu et al., 2014; Pandey et al., 2015). In the present study, we used the established AD *in vitro* model in APPswe cells and found through apoptotic and cytoactive analyses that Aβ led to high apoptotic ratios of neurons, accompanied by the activation of the Bax/caspase-3 axis, whereas upregulation of miR-200a-3p rescued neural apoptosis and recovered the apoptotic caspase-3 pathway. In addition, miR-200a-3p relieved the production of Aβ_1-42_, manifesting that miR-200a-3p protected against the injury derived from the overexpression of APP in the neuroblastoma SH-SY5Y cell line. Importantly, when miR-200a-3p and BACE1 expression were collaboratively established, the inversed effects of BACE1 on miR-200a-3p function were observed in APPswe cells, indicating an increase in cell apoptosis, an increase in caspase-3 activities, and an overproduction of Aβ_1-42_ peptides. Collectively, our findings may illuminate a potential mechanism indicating that miR-200a-3p contributed to the neuroprotective effects in AD *via* the regulation of BACE1.

In addition to the Aβ pathology, the role of tau hyperphosphorylation is another widely appreciated etiology in AD development. The tau protein is encoded by the *MAPT* gene, which consists of six isoforms in the central nervous system (CNS) generated by alternatively splicing and containing numerous sites that can be phosphorylated *via* the activity of various enzymes, including PKA, CDK5, GSK3β, and MAPK (Lee and Leugers, 2012). The development of NFTs is composed of three stages involving phosphorylated tau proceedings, preneural NFTs (pre-NFTs), intraneural NFTs, and extraneural NFTs. Phosphorylation epitopes at serine 199, 202, and 409 are associated with pre-NFT stages, whereas serine 396, 404, and threonine 231 occur in intraneuronal NFT stages (Kimura et al., 1996). The phosphorylation of tau at serine 396 and 404 sites was observed in early and late stages of AD and has been demonstrated to have greater preference in the earliest formation of NFTs, whereas the serine 214, serine 202, threonine 205 sites have been shown to be mostly associated with mature NFTs (Mondragon-Rodriguez et al., 2014). PKA is an important kinase that phosphorylates multiple sites of the tau protein, including serine 214, 396, and 356, alone or by sequential phosphorylation *via* cooperation with other kinases, such as GSK3β or CDK5, in the progression of AD (Jensen et al., 1999; Ksiezak-Reding et al., 2003; KyoungPyo et al., 2004; Wang et al., 2007). Essentially, prephosphorylated tau by PKA has been suggested to be the better substrate than the phosphorylation by other kinases, such as CDK5 and GSK3β (Liu et al., 2006). Thus, we chose the most representative phosphorylation sites of tau involved in AD and also PKA-preferred, such as serine 396, serine 214, serine 356, serine 202, and threonine 205, to perform our research.

As demonstrated in the present study *via* miRNA target prediction and validation using a dual-luciferase reporter assay, PRKACB, the catalytic units of PKA, was also identified as a direct target of miR-200a-3p, which negatively regulates the expression of PRKACB, thereby inhibiting PKA protein expression and activation. Moreover, miR-200a-3p demonstrated a consistent inhibitory effect on the phosphorylation of tau at serine 202/threonine 205 (AT8) in APPswe cells, including the AKT-preferable phosphorylation site serine 214, 396, and 356 (Jensen et al., 1999; Ksiezak-Reding et al., 2003; KyoungPyo et al., 2004). These observations provide further evidence of the miR-200a-3p-modulated pathological alterations of tau in AD. Moreover, our investigation showed that PKA overexpression blocked the inhibitory effects of miR-200a-3p on the modulation of tau phosphorylation at the examined phosphorylation sites of tau, including the epitopes that were mainly phosphorylated by PKA, *in vitro*. Taken together, we propose the existence of a regulatory event between miR-200a-3p and PKA that could help explain the mediation of tau hyperphosphorylation leading to the pathology of AD.

## Conclusions

**Figure 6:**
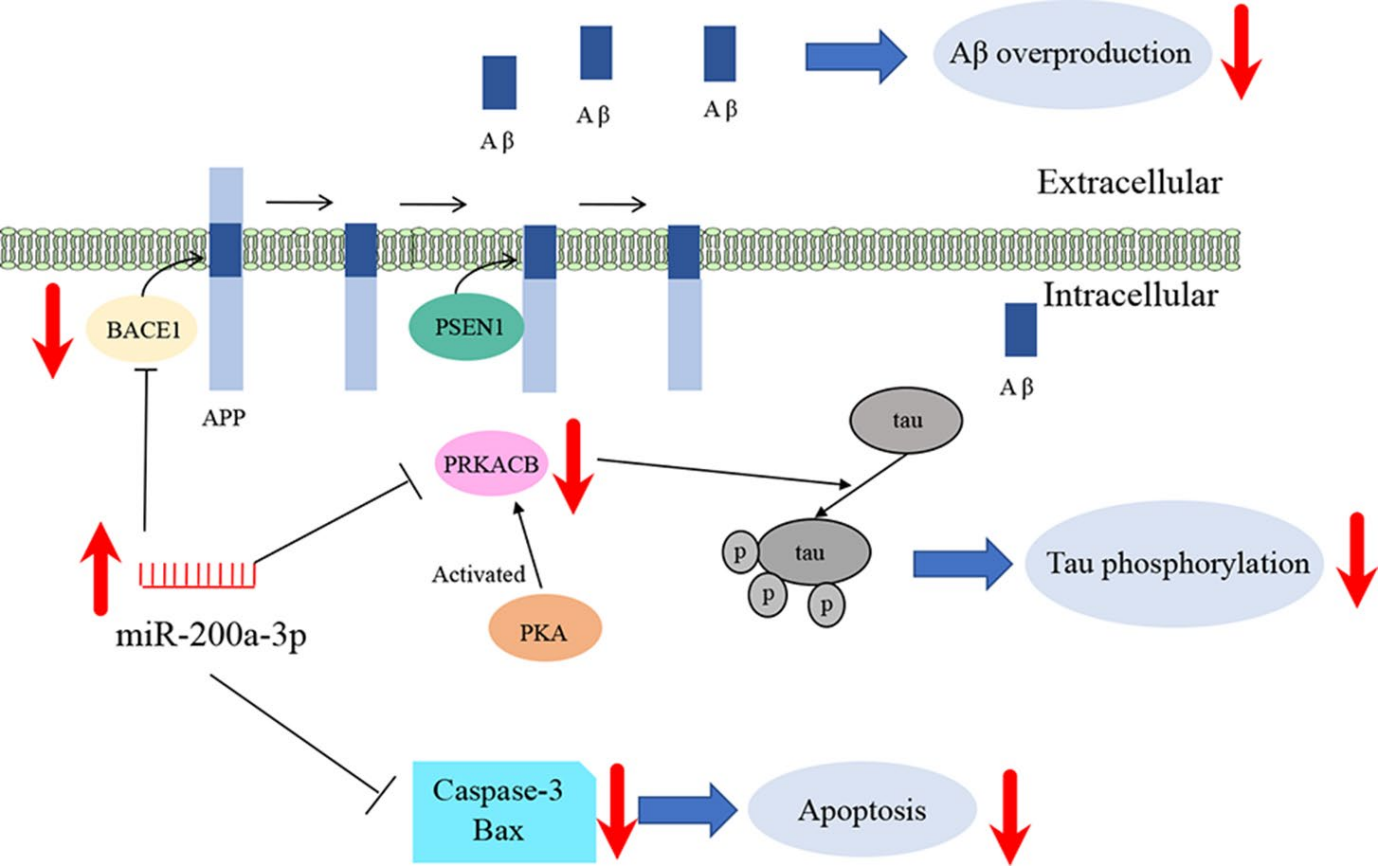
Schematic representation of miR-200a-3p neuroprotective effects in APPswe cells.

In summary, our study suggests that miR-200a-3p is implicated in AD progression and can exert neuroprotective effects against Aβ-induced toxicity by two possible mechanisms: first, by directly and/or indirectly inhibiting the overproduction of Aβ *via* suppressing the expression of BACE1 and second, by simultaneously decreasing the hyperphosphorylation of tau through attenuating the expression of PKA (**Figure 6**). In addition to this study, further mechanisms need to be explored, particularly those associated with the regulatory axis of miR-200a-3p. In addition, local delivery of miR-200a-3p into certain brain areas, such as the cortex and hippocampus, *via* proper and novel carriers may also shed a light on preventing detrimental effects of miRNA treatment in AD.

## Ethics Statement

The animals had *ad libitum* access to food and water at stable room temperature and humidity environment according to the Guide for the Care and Use of Laboratory Animals. The experiment was proved by the Ethical Committee of the Institute of Medicinal Biotechnology (IMB-D8-2018071102).

## Author Contributions

LW and JL carried out the experiments, analyzed the results, and wrote the manuscript. JL provided the plasma. QW, HJ, and LZ helped design the experiments, prepare the figures, analyze the data, and review the manuscript. RL and ZL designed the study and the experiments, interpreted the results, and wrote the manuscript. All authors have read and approved the final manuscript.

## Funding

This study was supported by the National Natural Science Foundation of China (No. U1803281 and 81673411), China; the Non-profit Central Research Institute Fund of Chinese Academy of Medical Sciences (2018RC350013), China; and the Chinese Academy Medical Sciences (CAMS) Innovation Fund for Medical Science (2017-I2M-1-016), China.

## Conflict of Interest Statement

The authors declare that the research was conducted in the absence of any commercial or financial relationships that could be construed as a potential conflict of interest.
