# Supplementary material for: MicroRNA-200a-3p Mediates Neuroprotection in Alzheimer-Related Deficits and Attenuates Amyloid-Beta Overproduction and Tau Hyperphosphorylation via Coregulating BACE1 and PRKACB
Source: Front Pharmacol. 2019 Jul 19;10:806. doi: 10.3389/fphar.2019.00806 (PMC6658613; doi:10.3389/fphar.2019.00806)
Supplement: Supplementary file 1 [file Presentation_1.pdf]

Supplementary Material

MATERIALS AND METHODS

Cell Apoptosis Assay

APPswe cells were transfected with BACE1 siRNA or PRKACB siRNA and accompanied transfected with miR-200a-3p mimics or their negative controls. The apoptosis was determined with FITC-Annexin V / propidium iodide (PI) Apoptosis Detection Kit (BD Biosciences; San Jose, CA). After 48 h, the cells were trypsinized and washed by PBS. Then, the cells were collected and resuspended in binding buffer and subsequently mixed with 5 μL Annexin V and 10 μL PI for 15 min in the dark. The ratio of apoptosis was analyzed by a FACSCalibur flow cytometry (BD Biosciences).

Statistical Analysis

Data are represented as the mean ± SEM. $P < 0.05$ was considered statistically significant. Comparisons were performed using one-way ANOVA, followed by appropriate post-hoc testing to analyze the differences between groups.

RESULTS

The Neuroprotective Effects of miR-200a-3p were in a Decreased Tendency When BACE1 and PRKACB were Knockdown

When we transfected BACE1 siRNA accompanied with miR-200a-3p mimics in APPswe cells, the effects of miR-200a-3p on reducing apoptotic ratios were shown in a decreased tendency. The same results were found in APPswe cells co-transfected with PRKACB siRNA and miR-200a-3p. Due to no significance was calculated, we speculated that the neuroprotective effects of miR-200a-3p on cell apoptosis might be associated with the regulation of BACE1 and PRKACB (**Supplementary Fig. 1**).

SUPPLEMENTARY FIGURES

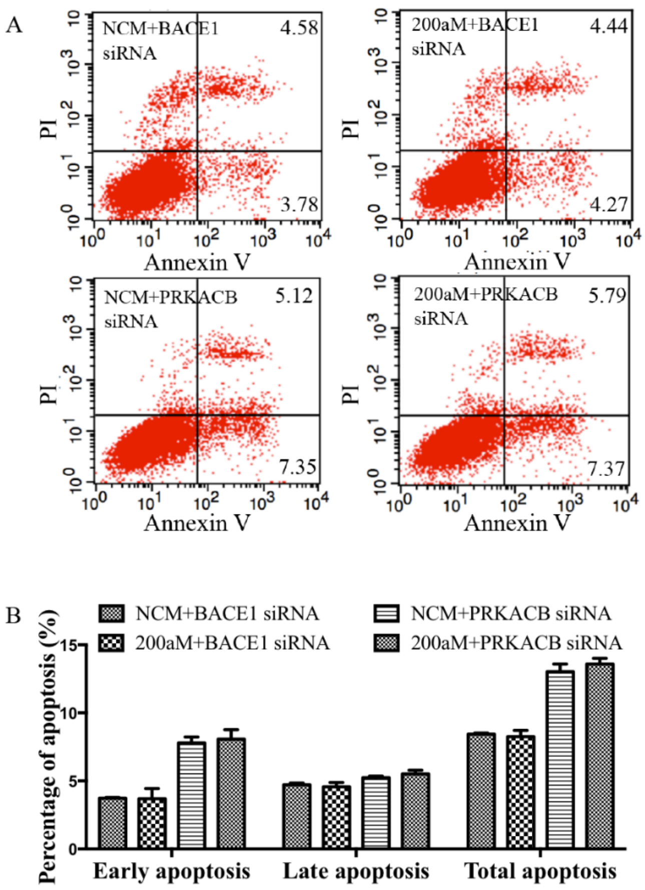

Supplementary Figure 1. miR-200a-3p neuroprotection was in a decreased tendency when BACE1 and PRKACB were knockdown. Effects of miR-200a-3p mimics (200aM), BACE1 siRNA, and PRKACB siRNA on apoptosis of APPswe cells detected using flow cytometry assay (A) and demonstrated by quantitative analysis (B). *n*=3. Data are shown as the mean ± SEM.
